# On a new diatomyid (Rodentia, Mammalia) from the Paleogene of south-east Serbia, the first record of the family in Europe

**DOI:** 10.1007/s12549-017-0301-4

**Published:** 2017-11-23

**Authors:** Zoran Marković, Wilma Wessels, Andrew A. van de Weerd, Hans de Bruijn

**Affiliations:** 1 Natural History Museum in Belgrade, Njegoševa 51, Belgrade, 11000 Serbia; 2 Department of Earth Sciences, Utrecht University, Heidelberglaan 2, P.O. Box 80021, 3508TA Utrecht, the Netherlands

**Keywords:** Rodentia, Diatomyidae, Oligocene, Serbia, Biogeography

## Abstract

A new diatomyid genus and species, *Inopinatia balkanica*, from the early Oligocene of south-east Serbia is described, and the affinities between the Diatomyidae and Ctenodactylidae are discussed. *Inopinatia balkanica* nov. gen. nov. sp. seems to have retained its deciduous teeth throughout life just as all other species of the family. The only other diatomyid described from outside south-east Asia which is *Pierremus explorator* López-Antoñanzas, 2010 is transferred to the thryonomyid species *Paraphiomys knolli* López-Antoñanzas and Sen, 2005.

## Introduction

**Fig. 1:**
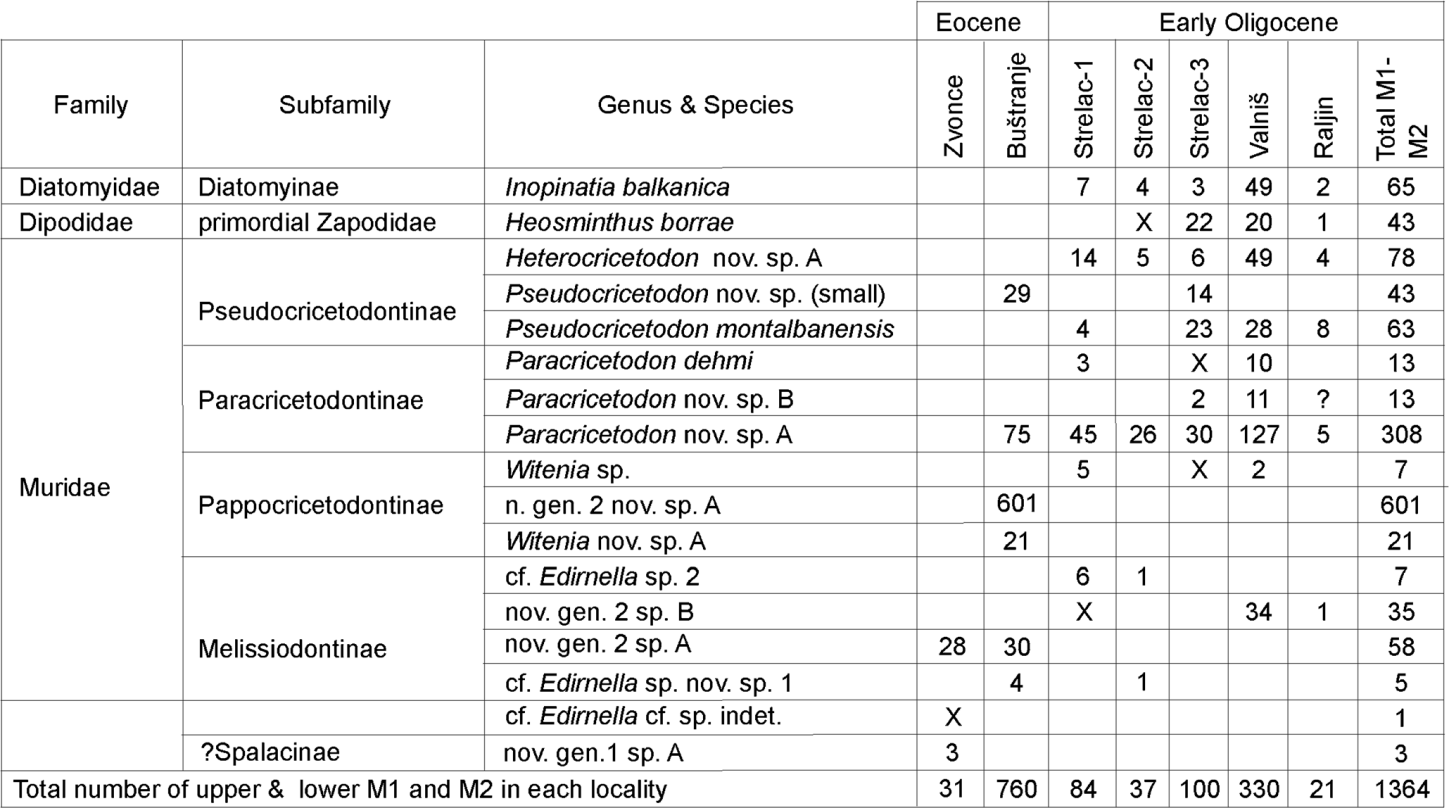
Distribution of rodent species in the localities of south-east Serbia

During the ongoing research of the Natural History Museum of Belgrade, upper Eocene and lower Oligocene non-marine deposits in basins of southern Serbia were investigated for the presence of fossil mammals. Early Oligocene rodent faunas were discovered in 2010 by Zoran Marković and Miloš Milivojević (Natural History Museum) in the Babušnica-Koritnica basin. The discovery was soon followed by collecting campaigns in close cooperation with Hans de Bruijn and Wilma Wessels of the University of Utrecht. At present, two rodent faunas of late Eocene age and five of early Oligocene age have been found. For the geological setting, the geographical position and an overview of fossil content of these sites we refer to Bruijn et al. (in press). The distribution of rodent taxa in the faunas is shown in Fig. 1.

In this paper, we will describe a new genus and species of the Diatomyidae from the early Oligocene localities in the Babušnica-Koritnica basin. It is the first occurrence outside of Asia of this rodent family, and the Serbian fossils are among the oldest representatives known. Prior to the findings in southern Serbia, fossils of this family were known only from the Oligocene and Miocene of Asia. The extant diatomyid *Laonastes aenigmamus* Jenkins et al., 2005 was discovered in Laos in 2004. A few years later, Dawson et al. (2006) included this new animal in the Diatomyidae.

The discovery of a new diatomyd genus in Serbia, a family so far know only from Asia, suggests a biogeographic pattern similar to that of the few findings of larger mammals in upper Eocene and Oligocene deposits in the Balkan countries of Bulgaria and Romania. These show strong taxonomic affinities to Asian species while they are absent in the well-known Central and Western European faunas.

The Eocene-Oligocene transitional interval showed major changes in climate, palaeogeography and biogeography. In Central and Western Europe, these changes are reflected in the mammalian faunal turnover known as the “*Grande Coupure*”*.* Mammal families that arrived in Central or Western Europe after the “*Grande Coupure*” were already present in Serbia in the late Eocene. However, the diatomyid family did not reach Central or Western Europe.

## Methods

The teeth that will be described and discussed have been collected by wet-screening fossiliferous matrix from Valniš, Strelac-1, Strelac-2, Strelac-3 and Raljin on a set of stable sieves in the field (finest mesh used is 0.5 mm.). The residue obtained has been rewashed on a vibrating set of sieves (Marković and Milivojević 2010). In order to reduce the concentrate even further, it has been treated with diluted acetic acid in the laboratory. The large amount of gypsum crystals in the concentrate from Raljin has been removed by heating the residue to 150 °C for several hours. The fractions larger than 0.65 mm have been sorted under a microscope. The measurements of the teeth have been taken with a Leitz Ortholux measuring microscope with mechanical stage and measuring clocks. All specimens are figured as left ones. If the original is from the right side, this is indicated by underlining its number on the figure. Lower case letters refer to the lower dentition, upper case letters refer to the upper dentition. Abbreviations for measurements and descriptions are N—number of specimens, R—range of measurements, L—length, W—width, sin—sinistral and dext—dextral.

Abbreviations and terminology used in the description of the microstructure of enamel are EDJ—enamel-dentine junction, HSB—Hunter-Schreger band, inclination—angle between the HSB and the normal to the EDJ, PI—portio interna, PE—portio externa, OES—outer enamel surface, PLEX—external enamel layer without prisms, IPM—inter prismatic matrix, radial enamel—enamel with parallel prisms that are at right angles to the EDJ and BRLE—basal ring of lamellar enamel. The fossil assemblages from southeastern Serbia are housed in the Natural History Museum in Belgrade (Serbia). A representative set of casts of rodents is kept in the collection of the department of Earth Sciences of Utrecht University, the Netherlands. The locality codes of the Natural History Museum of Belgrade and abbreviations used for of the localities are 024 for Strelac-1 (STR-1), 025 for Strelac-2 (STR-2), 026 for Strelac-3 (STR-3), 027 for Valniš (VA) and 028 for Raljin (RA).

## Taxonomy

### Introduction

Our new genus and species of the Diatomyidae is among the oldest records and the first solid record outside of Asia of the family. However, as will be discussed below, *Inopinatia balkanica* nov. gen. nov. sp. shares some basic dental characters with the Ctenodactylidae.

**Fig. 2:**
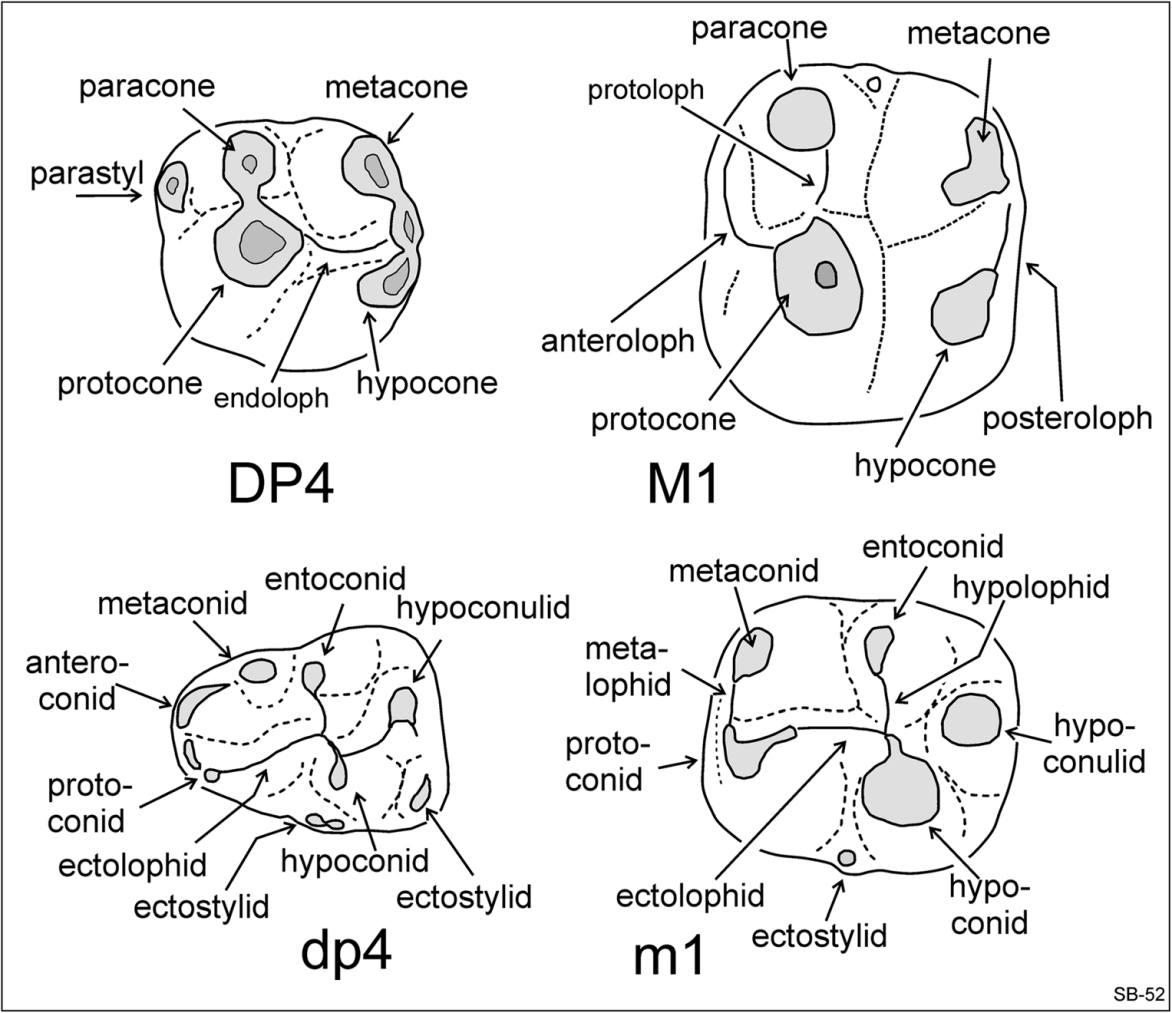
Cusp terminology for the cheek teeth of *Inopinatia* nov. gen.

The reconstruction of the dentition of rodents with premolars on the basis of isolated teeth often introduces uncertainties in recognising the tooth position in the jaw (Flynn et al. 1986). In the case of *Inopinatia* nov. gen., this problem is pertinent, because the genus closest in dental morphology is the diatomyid *Fallomus*, a genus containing three species that are all exclusively known by isolated teeth. The terminology of the cusps in the descriptions below is shown in Fig. 2.

*Inopinatia* nov. gen.

**Type species:** *Inopinatia balkanica* nov. sp.

**Type locality:** *Valniš*

**Age:** Early Oligocene

**Derivation nominis:** from the Latin word inopinatus meaning unexpected

**Diagnosis:** *Inopinatia* is a large diatomyid with an incipiently bi-lophodont pattern of the cheek teeth. Endoloph in upper molars absent, ectolophid in lower molars weak. DP4 and dp4 large and molariform. Deciduous teeth are retained throughout life. DP4, M1 and M2 with three roots. Enterostyle absent in the upper cheek teeth. The m1 and m2 have three roots, two roots anteriorly and one posteriorly, a very large hypoconulid and a small ectostylid. The dp4 has an ectostylid behind the hypoconid. Microstructure of the enamel of the incisors multiserial. Molar enamel is S-type. Upper incisor without sulcus.

**Differential diagnosis:** *Inopinatia* differs from *Fallomus* and *Marymus* in having M3/m3 that are larger instead of smaller than the M2/m2 and in retaining the endoloph of the DP4 and the ectolophid of the m1, m2 and m3. In *Inopinatia*, the large hypoconulid is the highest cusp in unworn dp4, m1, m2 and is never reduced as in some m1 and m2 of *Fallomus* and *Marymus*.

**Fig. 3:**
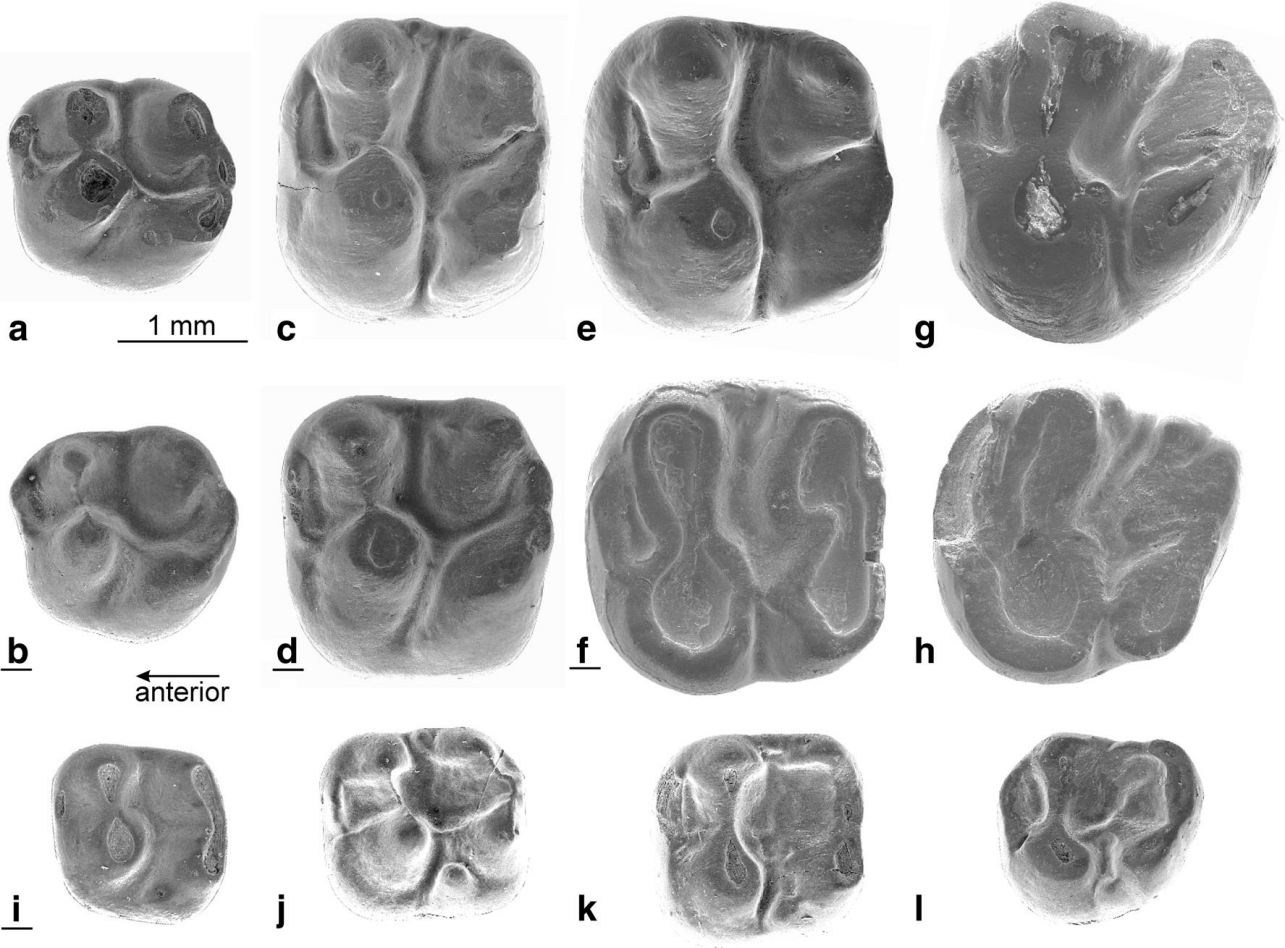
Upper cheek teeth of *Inopinatia balkanica* nov. sp. from Valniš. **a** DP4 (VA-108). **b** DP4 (VA-115). **c** M1 (VA-143). **d** M1 (VA-152). **e** M2 (VA-142). **f** M2 (VA-152). **g** M3 (VA-161). **h** M3 VA-163*. Fallomus razae* from its type locality Pazbogi Nala GSP-417. **i** DP4. **j** M1. **k** M2. **l** M3

**Fig. 4:**
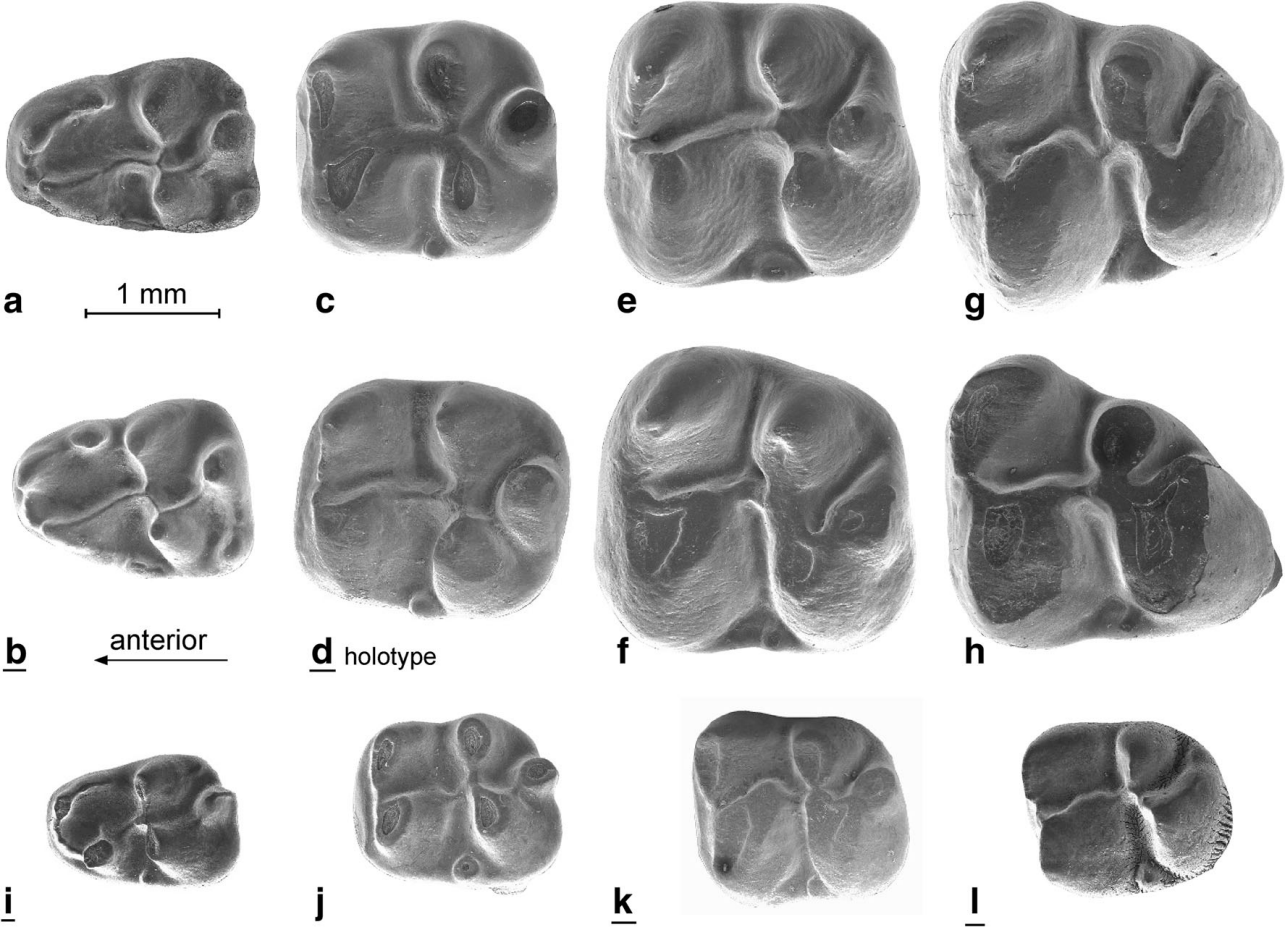
Lower cheek teeth of *Inopinatia balkanica* nov. sp. from Valniš. **a** dp4 (VA-204). **b** dp4 (VA-195). **c** m1 (VA-204). **d** m1 (VA-213). **e** m2 (VA-221). **f** m2 (VA-222). **g** M3 (VA-241). **h** M3 VA-243. *Fallomus razae* from its type locality Pazbogi Nala GSP-417. **i** dp4. **j** m1. **k** m2. **l** m3

*Inopinatia balkanica* nov. sp.

(Fig. 3a–h, Fig. 4a–h)

**Holotype:** m1 dext. (Fig. 4d, no. VA-213)

**Type locality:** Valniš

**Age:** Early Oligocene

**Material and measurements:** see Tables 1 and 2.

**Table 1:** Measurements of cheek teeth of *Inopinatia balkanica* nov. sp. from the type locality Valniš

	Length (mm)			Width (mm)		
**Valniš**	**Range**	**Mean**	**Number**	**Mean**	**Range**	**Number**
DP4	1.51–1.75	1.62	13	1.57	1.48–1.67	13
M1	1.87–2.13	1.97	12	2.01	1.80–2.27	12
M2	2.10–2.33	2.22	11	2.30	2.20–2.42	11
M3	2.08–2.46	2.24	5	2.24	2.09–2.53	5
dp4	1.55–1.84	1.69	11	1.24	1.08–1.32	13
m1	1.83–2.09	1.95	12	1.71	1.60–1.78	12
m2	2.10–2.32	2.20	10	2.07	1.87–2.22	9
m3	2.26–2.59	2.39	8	2.09	1.94–2.31	9

**Table 2:** Measurements of the cheek teeth of *Inopinatia balkanica* nov. sp.from the localities Strelac-1, Strelac-2 and Raljin

	Length (mm)			Width (mm)		
**Strelac-1**	**Range**	**Mean**	**Number**	**Mean**	**Range**	**Number**
DP4		1.62	1	1.54		1
M1	1.91–2.03	1.97	2	1.88	1.79–1.97	2
M3	2.15–2.19	2.17	2	2.12	2.08–2.16	3
m1	1.77–1.95	1.86	3	1.71	1.64–1.78	3
m2	2.09–2.33	2.24	2	1.98	1.85–2.11	2
**Strelac-2**						
DP4		1.59	2	1.58		2
M1	1.89–2.02	1.96	2	2.05	2.02–2.08	2
dp4	1.75–1.75	1.75	2	1.50	1.35–1.44	2
m1	2.01–2.02	2.02	2	1.77	1.76–1.77	2
**Raljin**						
M3		2.31	1	2.13		1
m1		1.95	1	1.75		1

### Description of the upper cheek teeth (Fig. 3a–h)

The DP4 is a molariform tooth. The short anteroloph connects the labial side of the protocone with the anterior side of the paracone in most DP4, but in some others, it is very short and developed as a parastyle. There is no wear facet on the anterior side, so *Inopinatia* did not have a DP3. The protocone and the paracone are situated very close to one another and are fused at an early wear stage. The hypocone and the metacone, the highest cusps in fresh specimens, are connected by the posteroloph. In some DP4, this posteroloph is somewhat wider halfway between the hypocone and the metacone. The protocone is connected to the hypocone by an oblique endoloph. The DP4 has three roots.

The M1 and M2 have the same morphology, but the M1 is somewhat smaller than the M2. Since there is a minor overlap in the size of these teeth, we cannot exclude that a small M2 has been listed as an M1 or the other way around. The anteroloph of the M1 and M2 is lower than the protoloph and becomes incorporated into that ridge at an advanced wear stage. The valley between the transverse protoloph and the fused metaloph /posteroloph is open lingually as well as labially, but low remnants of the endoloph and a tiny mesostyle are present in some specimens. The short incomplete metaloph that is directed towards the protocone, is clearly distinguishable from the posteroloph in fresh specimens. The posteroloph connects the hypocone with the metacone in most specimens, but there may be a narrow notch that isolates the metacone. The M1 and M2 have three roots each.

The M3 is large, and the morphology of the M3 is similar to that of the M1 and M2, but compared to the M1-M2, the anteroloph is somewhat stronger; the metacone and hypocone are situated closer together, and the incomplete metaloph is more clearly directed towards the protocone.

### Description of the lower cheek teeth (Fig. 4a–h)

The dp4 has three cusps on its anterior part (presumably the protoconid, anteroconid and metaconid); these are incorporated into a loop that includes the ectolophid. The relative height and size of these three cusps shows considerable individual variation. The oblique ectolophid connects the protoconid with the hypolophid. Among the three cusps of the talonid, the hypoconid is usually the smaller and the hypoconulid the larger. Seven out of the nine dp4 from the type locality have a small ectostylid-1 between the protoconid and hypoconid, and all nine specimens have an ectostylid-2 behind the hypoconid. The dp4 has two roots.

The m1 and m2 have very similar dental patterns in showing five major cusps (protoconid, metaconid, hypoconid, entoconid and hypoconulid). Among these, the hypoconulid is the highest in unworn teeth. Our distinction between m1 and m2 is mainly based on the greater width of the m2. The slightly forwardly directed metalophid determines the anterior limit of the lingual part of the occlusal surface. The short transverse hypolophid connects to the ectolophid in front of the hypoconid. Although the ectolophid is much lower than the main cusps, it is well developed in all m1 and m2. A small ectostylid is present in the m1 and m2. The large high hypoconulid is an isolated cusp in some specimens, but in others, it is part of the posterolophid. The m1 and m2 have three roots each; a very wide one is posteriorly that may or may not have been split near the tip, and the two are anteriorly.

The m3 is large and similar to the m1 and m2, but its hypoconulid is incorporated into a short, wide, posterolophid, and the ectolophid is weaker than in the m1 and m2. The roots have not been preserved in any of the m3.

### The microstructure of the tooth enamel

**Fig. 5:**
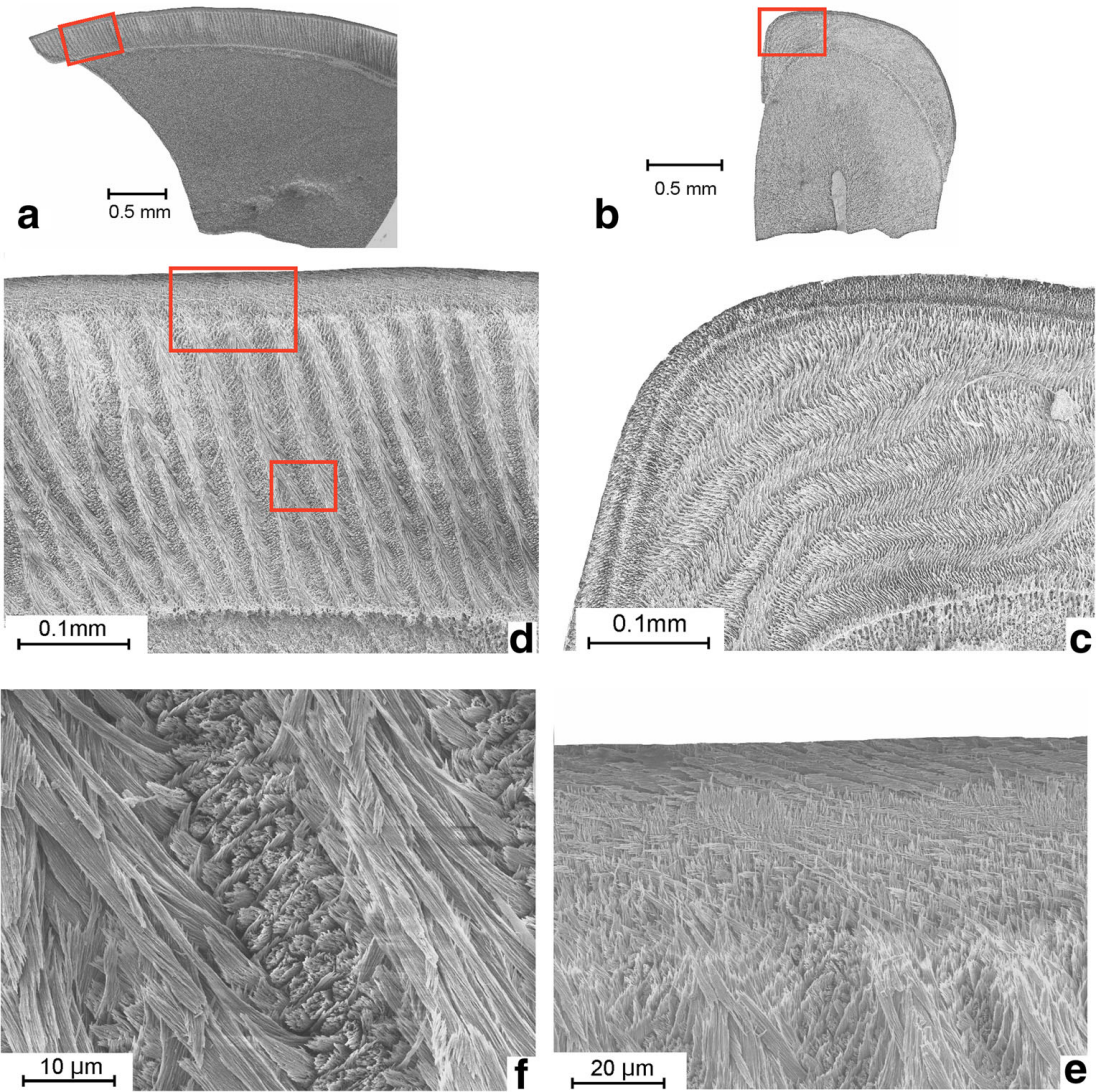
Sections of the upper incisor of *Inopinatia balkanica* nov. sp. from Valniš. **a** Sagittal section. **b** Transverse section. **c** Detail of b. **d** Detail of a. **e** Detail of d (upper square). **f** Detail of d (smaller square)

**Fig. 6:**
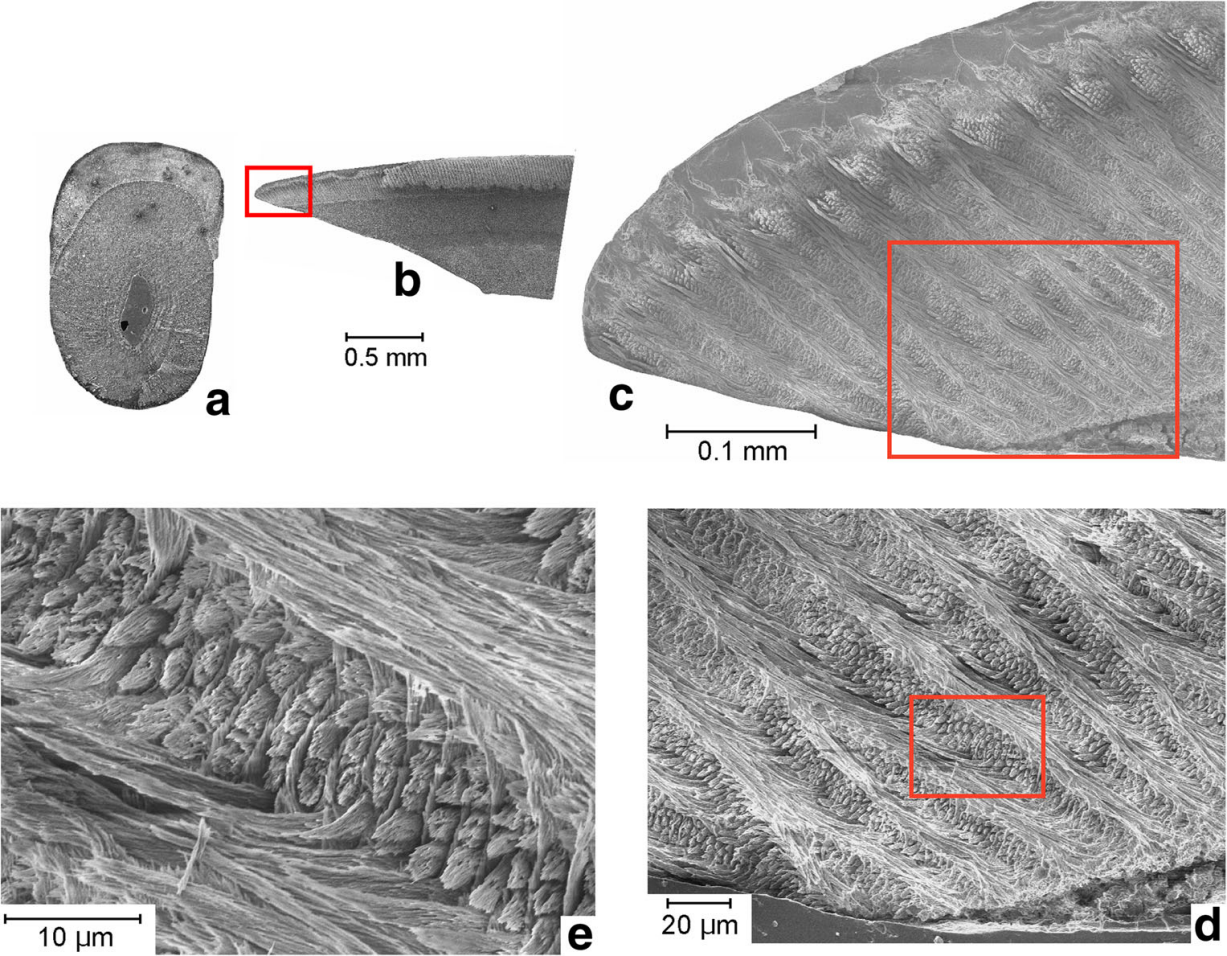
Sections of the lower incisor of *Inopinatia balkanica* nov. sp. from Valniš. **a** Transverse section. **b** Sagittal section. **c** Detail of b. **d** Detail of c. **e** Detail of d

Incisor material is relatively limited and poorly preserved, this in particular so in Valniš. Since crocodile teeth are abundant in that deposit, we think that the fragmentation of the rodent incisors may be due to digestion by these animals. This interpretation is supported by the poor preservation of bones, which are represented by many small, often shiny polished, fragments. The sagittal and cross sections shown were prepared by using two pieces of the same upper and lower incisor. The identification of isolated incisors is usually problematic, but since the species recognised in these assemblages are, with the exception of *Inopinatia*, either Muridae or Dipodidae—two families that have an uniserial enamel structure—we are sure that the incisors with multiserial enamel belong to *Inopinatia*. Remarkable is the similarity in shape of the cross-sections of the lower and upper incisors of this rodent (Figs. 5b and 6a).

### The upper incisor (Fig. 5)

The exterior surface is almost smooth. The thick enamel extends rather far over the internal and external sides and shows a prominent almost rectangular bend mesially. The quotient of the antero-posterior and mesio-distal section is about 2. The thickness of the enamel is about 260 μm in the sagittal plane and the PE is thin, occupying a little over 10% of the total. The boundary between the PI and the PE is sharp. The HSB make an angle of about 20° with the normal on the EDJ. The number of prisms in the often anastomosing HSB is mostly three and the cross section of the prisms is oval. Locally, there seems to be a very thin layer of internal radial enamel. The PLEX is absent, and the IPM makes an angle of about 90° with the prisms.

### The lower incisor (Fig. 6)

The exterior surface of the enamel of the rather thick lower incisor is basically smooth with a very vague crenulation. The enamel extends relatively far over the internal and external sides and shows a prominent, almost rectangular bend mesially, which makes the *Inopinatia* incisor readily recognisable. The quotient of the antero-posterior and the mesio-distal section is about 1.5. The thickness of the enamel is about 175 μm with a PE occupying about 30% of the total thickness in the central part of the tooth. The often anastomosing HSB makes an angle of about 30° with normal on the EDJ. The number of prisms per HSB is mostly three and their cross section is oval. Internal radial enamel and PLEX are absent. The IPM in the prisms make about right angles with the prisms. The boundary between the PI and the PE is in many places not clear due to crystallisation, but seems to have been sharp originally.

### The cheek teeth (Fig. 7)

The portio interna of the base of the enamel of the cheek teeth shows a rather high ring of lamellar enamel with thick transverse HSB (about 6 prisms/HSB). The portio externa of this ring consists of a thin layer of radial enamel. Towards the occlusal surface, the lamellar enamel grades gradually into radial enamel (type S of von Koenigswald 2004).

**Fig. 7:**
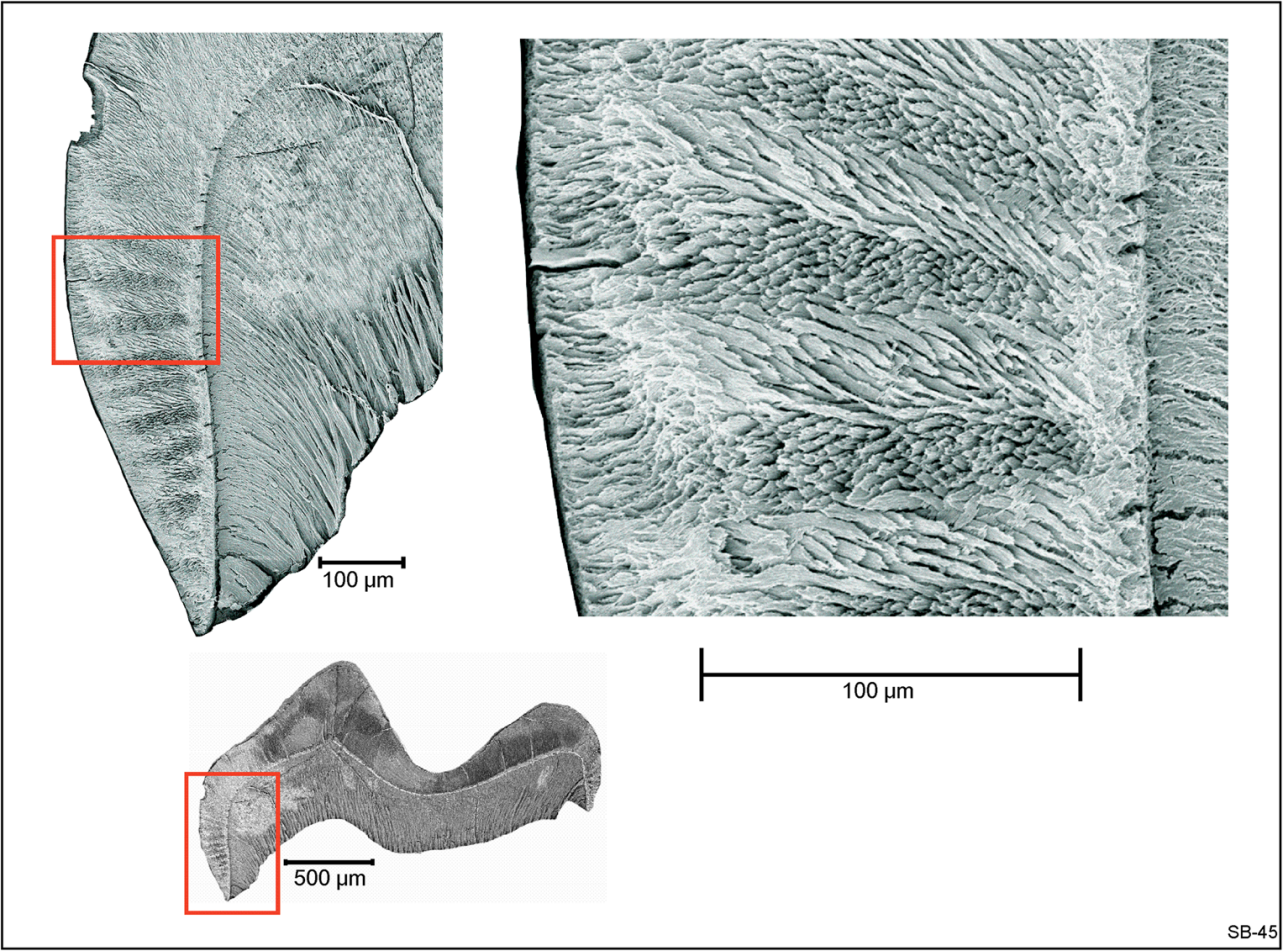
Section through a molar of *Inopinatia balkanica* nov. sp. from Strelac-1

## Comparison and discussion

The morphology of the *Inopinatia balkanica* cheek teeth shares characteristics with early members of the Ctenodactylidae such as *Butomys* Dashzeveg and Meng, 1998, *Mergenomys* Dashzeveg and Meng, 1998 and *Ulanomys* Dasheveg, 1990 from the Eocene of Mongolia.

Features shared by *Inopinatia* and the Ctenodactylidae are (1) increase in size from M1/m1 through M3/m3, (2) a longitudinal ridge in the DP4 and in the lower cheek teeth, (3) a large hypoconulid in the dp4, m1 and m2, (4) a (short, incomplete) metaloph that is directed towards the protocone in the upper molars and (5) multiserial incisor enamel.

*Inopinatia* differs from the Ctenodactylidae by having an incipiently bi-lophodont pattern, m1 and m2 with more than two roots and by retaining the DP4 and dp4 throughout life. The latter characteristics differentiate early Diatomyidae such as *Fallomus* Flynn et al., 1986 (Oligocene of Baluchistan) illustrated in Fig. 3i–l and 4i–l and *Marymus* Flynn, 2007 (early Miocene of Pakistan) and *Inopinatia* nov. gen. from the Ctenodactylidae.

Features shared by *Inopinatia* and the Diatomyidae are (1) incipient bi-lophodonty of the cheek teeth, (2) absence of the longitudinal ridge in the upper molars, (3) retention of the deciduous teeth throughout life, (4) presence of more than two roots in the m1 and m2 and (5) presence of a small ectostylid in the lower molars. Since all these features are interpreted as synplesiomorph, we allocate *Inopinatia balkanica* to the Diatomyidae.

The two morphs distinguished by Flynn et al. (1986) and by Marivaux and Welcomme (2003) among the premolars of *Fallomus* that were thought to mark deciduous from permanent teeth, seem to be within the range of the individual variation of the milk teeth. This means that all known species of the Diatomyidae have retained the deciduous teeth throughout life. If this conclusion is correct, the diagnoses of the family as given by Mein and Ginsburg (1997), Marivaux and Welcomme (2003), Dawson et al. (2006) and Flynn (2007) will have to be revised, because the reference in these studies to a large P4/p4 seems to apply to a DP4/dp4.

The presence of an interdental wear facet on the anterior side of some of the “P4” of *Fallomus razae* and of *Fallomus ginsburgi* reported by Flynn et al. (1986) and Marivaux and Welcomme (2003), suggesting the presence of a residual DP3, could not be confirmed. At this stage, we consider it premature to adapt the diagnoses of Diatomyidae*.*

## Biogeography

With about 11% of the first and second molars, *Inopinatia balkanica* is common (see Fig. 1) in the five early Oligocene rodent faunas from the Babušnica-Koritnica basin.

The discovery in Serbia of a rodent genus completely absent in western and central Europe, but closely related to genera from Asia is in line with the biogeographic pattern observed earlier in the Paleogene fossil larger mammals from the Balkan region (Petronijević and Thenius 1957; Nikolov and Heissig 1985; Heissig 1990; Böhme et al. 2013). We can therefore safely exclude the possibility that our new genus from Serbia is a case of convergent evolution and not a true diatomyid.

The only other record of a diatomyid from outside is *Pierremus explorator* Lopez Antoňanzas, 2010 from the Lower Miocene Dam Formation in Saudi Arabia. This species is based on a single premolar which, in our opinion, belongs to the thryonomyid *Paraphiomys knolli* López-Antoñanzas and Sen, 2005, a species described from the same fauna. The allocation by López-Antoňanzas (2010) of this single premolar from As Sarrar to the Diatomyidae is based on two synapomorphies: “A tri-lophodont dental pattern and the position of the entoconid, which is displaced with respect to the hypoconid”. In our opinion, this is inadequate evidence for its familial allocation, because this morphology occurs in a number of other rodent families, for instance in the lower premolar of the thryonomyids *Paraphiomys* Andrews, 1914 and *Kirtharia* de Bruijn, 1986 (= *Kochalia* de Bruijn and Hussain, 1985) and in the first lower molar of the early spalacids *Debruijnia* Ünay, 1999 and *Vetusspalax* de Bruijn et al., 2013. Reasons for not including the single premolar in *Paraphiomys knolli* from the same locality are not provided by López-Antoňanzas.

**Fig. 8:**
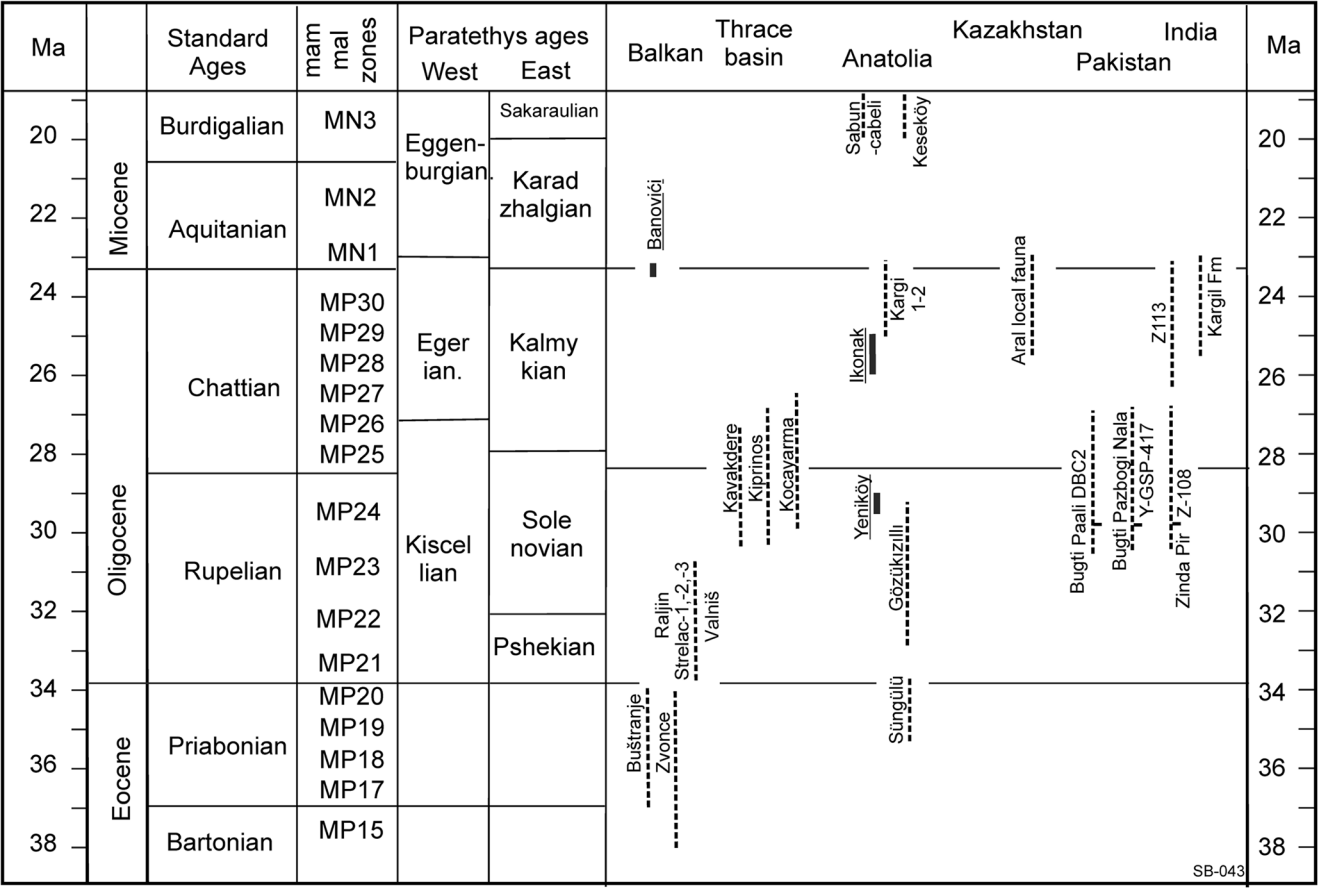
Stratigraphic scheme illustrating the stratigraphic position of the faunas discussed. Three faunas are indicated with a solid bar; these have age control from geomagnetic or radiometric studies

Diatomyid rodents are common in the Oligocene faunas of Pakistan, where, in the Paali Nala DBC2 locality of the Bugti Hills, three species occur (Marivaux and Welcomme 2003). The age range of this site is probably late Rupelian to early Chattian (see Fig. 8). Flynn (2007) described another low-crowned genus and species *Marymus,* from the upper Oligocene site Z113 of the Chirtawata Formation of the Zinda Pir Dome (Fig. 8).

Kumar et al. (1996) and Nanda and Sahni (1998) report a few dental elements of a high crowned diatomyd species, *Fallomus ladakhensis,* from the upper Oligocene Kargil Formation in India (Fig. 8), which occurs together with a low-crowned diatomyd species*.*

High crowned diatomyid specimens were also found in Thailand in deposits of probable late Oligocene age and allocated to *Fallomus ladakhensis* (Marivaux et al. 2004).

Oligocene faunas from localities situated in-between south-east Serbia and India are known from the Thrace basin, Anatolia and from Kazakhstan (Fig. 8). However, in neither of these Diatomyidae are present, nor are Oligocene diatomyid rodents known in the Oligocene of Central Asia.

Unfortunately, the ages assigned to the faunas discussed above are based on biostratigraphic correlations, resulting in relatively long ranges for their age estimates. An additional problem is that the fauna successions in these areas are insufficiently known.

The late Rupelian-early Chattian Bugti Hill faunas (Marivaux and Welcomme 2003) and the early Oligocene rodent faunas from Serbia share only the cricetid genus, *Pseudocricetodon*, and the presence of a diatomid. *Pseudocricetodon* reached the Bugti area using a land connection between its Asian heartland and the Indian continent (fig. 4 in Métais et al. 2009) and migrated into the Balkan using the Iranian-Anatolian route. Diatomyid rodents probably used the same land connections to migrate from Asia into India and the Balkan.

At this moment, we have only two windows into the Paleogene rodent succession of Serbia: the Eocene assemblages from Zvonce and Buštranje (see Fig. 1) and the robust Oligocene assemblages from Strelac-1, -2 and -3, Valniš and Raljin (Bruijn et al., in press). The faunas from these two time slices are very different; the Oligocene assemblages contain the first record of diatomyid and dipodid rodents. Because we have only two windows, it is not known whether the faunal influx from Asia into Serbia was a gradual process over a very long period or restricted to a well-delimited event. If the latter option is correct, this event could be the same early Oligocene climatic change that triggered the “*Grande Coupure”* of Western and Central Europe.

### **Conclusions**

The presence of the rodent family Diatomyidae in Serbia means a great range extension of this family, known before only from the Indian subcontinent and Asia. All known species of the Diatomyidae seem to have retained the deciduous teeth throughout life. The two morphs distinguished by Flynn et al. (1986) and by Marivaux and Welcomme (2003) among the premolars of *Fallomus* that were thought to mark deciduous from permanent teeth, seem to be within the range of the individual variation of the deciduous teeth. This means that the diagnosis of the family will have to be adapted accordingly. The conclusion of Marivaux and Welcomme (2003) that Diatomyidae have a P3 is based on their observation of a wear facet on the anterior side of a P4 (in our opinion DP4). Since neither the DP4 of *Fallomus razae* from the type locality nor the DP4 of *Inopinatia balkanica* show a facet of the DP3, we conclude that the presence of this tooth in *Fallomys* is doubtful. The dental similarity of early diatomyids suggests that the ancestry of the Diatomyidae should be looked for among the Ctenodactyloidea, a conclusion that is in line with the micro-structure of the incisor enamel and the molecular evidence presented by Huchon et al. (2007). The family may well have originated in central Asia and not on the Indian subcontinent.
